# Coronary artery bypass surgery in high-risk patients

**DOI:** 10.1186/1468-6708-6-13

**Published:** 2005-08-26

**Authors:** Alper Sami Kunt, Osman Tansel Darcın, Mehmet Halit Andac

**Affiliations:** 1 Department of Cardiovascular Surgery, Harran University Research Hospital, Sanlıurfa, Turkey

**Keywords:** Coronary artery, high risk, off-pump, on-pump, surgery

## Abstract

**Background:**

In high-risk coronary artery bypass patients; off-pump versus on-pump surgical strategies still remain a matter of debate, regarding which method results in a lower incidence of perioperative mortality and morbidity. We describe our experience in the treatment of high-risk coronary artery patients and compare patients assigned to on-pump and off-pump surgery.

**Methods:**

From March 2002 to July 2004, 86 patients with EuroSCOREs > 5 underwent myocardial revascularization with or without cardiopulmonary bypass. Patients were assigned to off-pump surgery (40) or on-pump surgery (46) based on coronary anatomy coupled with the likelihood of achieving complete revascularization.

**Results:**

Those patients undergoing off-pump surgery had significantly poorer left ventricular function than those undergoing on-pump surgery (28.6 ± 5.8% vs. 40.5 ± 7.4%, respectively, *p *< 0.05) and also had higher Euroscore values (7.26 ± 1.4 vs. 12.1 ± 1.8, respectively, *p *< 0.05). Differences between the two groups were nonsignificant with regard to number of grafts per patient, mean duration of surgery, anesthesia and operating room time, length of stay intensive care unit (ICU) and rate of postoperative atrial fibrillation

**Conclusion:**

Utilization of off-pump coronary artery bypass graft (CABG) does not confer significant clinical advantages in all high-risk patients. This review suggest that off-pump coronary revascularization may represent an alternative approach for treatment of patients with Euroscore ≥ 10 and left ventricular function ≤ 30%.

## Introduction

Early mortality and morbidity represent clinical outcomes that have been used in many research models examining patients undergoing coronary artery bypass graft (CABG) surgery [1-6]. Studies utilizing these endpoints have provided valuable information for determining the indications for surgery, estimating the need for various resources and implementing quality control monitoring of surgeons and institutions. The European System for Cardiac Operative Risk Evaluation (EuroSCORE) used logistic regression analysis to identify and give appropriate weight to various risk factors related to in-hospital mortality in adult cardiac operations [7]. Standard EuroSCORE was first introduced in 1999 [8] as an additive system and has gained wide acceptance in Europe [9]. The logistic algorithm, which recently, became available [10], appears to be a better risk predictor of mortality and morbidity in CABG patients, especially among high-risk patients [11,12].

A EuroSCORE value >5 reflects a high level of risk in patients with coronary artery disease (CAD). Severe LV dysfunction represent another clinical outcome that has been reported to serve as an independent predictor of operative mortality in patiens undergoing CABG [13]. Coronary artery disease patients with reduced left ventricular function appear to benefit more from CABG than from medical therapy [14].

In high-risk CAD patients, surgical myocardial revascularization often produces poor results leading to significant mortality and morbidity [15]. Management of high-risk patients remains unclear. We describe our experience in the treatment of high-risk CAD patients undergoing on-pump versus off-pump surgery.

## Materials and methods

### Clinical data collection

The records of 86 consecutive high-risk patients who underwent primary isolated CABG at Harran University Research Hospital between January 2002 and December 2004 were reviewed retrospectively. The study was approved by the ethics committee of the Harran University Research Hospital, and informed consent was obtained from all patients. Patients were considered to be high-risk were included in the study if they had a preoperative Euroscore of ≥5 on admission to the hospital.

Preoperative and postoperative patient data were reviewed using registry databases, medical notes and charts. Forty patients underwent CABG using the off-pump technique while 46 patients were operated on using the conventional on-pump technique. Selection of either technique was done by the individual surgeon, and was based on his experience and preference. No randomization was involved in this cohort of patients.

### Operative technique

#### Anesthesia

All routine cardiac medications were continued up until the morning of surgery. After premedication with 5 mg intramuscular Midazolam and 0.18 mg/kg of intrathecal morphine diluted in 4 ml of serum physiologic solution for postoperative analgesia, anesthesia was induced using 0.3 mg/kg of etomidate, μg/kg of remifentanil and 0.6 mg/kg of rocuronium intravenously. After endotracheal intubation, desflurane (3–10%) and remifentanil 0.25–1.0 μg/kg/min in air/oxygen and rocuronium were given to maintain anesthesia.

#### On-pump technique

After the standard median sternotomy, aorta-right atrial cannulation and cardiopulmonary bypass were performed in on-pump patients. During cardiopulmonary bypass (CPB), hematocrit, mean arterial pressure, and pump flow were maintained at 20–30%, 50–80 mmHg, and 2.2–2.5 l/m^2^, respectively. Adequacy of tissue perfusion was monitored, as well as arteriovenous partial carbon dioxide difference (P_v-a _CO_2_), urine output, and base deficit. Patients were cooled to 32°C with moderate hypothermia. Desflurane-remifentanil anesthesia was administered during CPB. Revascularization procedures were performed with aortic cross-clamping. During myocardial ischemia antegrade cold hyperkalemic crystalloid cardioplegia was used (Plegisol^®^, Abbot Laboratories, IL, and USA). After completion of distal anastomosis, the proximal anastomosis was performed to the ascending aorta by using a side-biting clamp.

#### Off-pump technique

Left internal mammary artery and saphenous vein grafts were harvested for grafting for off-pump patients. To provide better access to lateral and posterior target vessels the pericardium was retracted by two or three deep sutures and two sponges were placed under the heart. Neither a heart stabilizer nor intraluminal shunts were used. Silicone snare sutures were placed proximal and distal to the anastomosis in order to provide a bloodless field. Remifentanil infusion and desflurane were discontinued at skin closure.

### Statistical analysis

All clinical data were expressed as mean ± standard deviation. Data processing and statistical analysis were performed using SPSS statistical software package for Windows. The student's t-test and chi-square test were used. A *p *value < 0.05 was considered statistically significant.

## Results

### Preoperative characteristics

The preoperative characteristics of patients are listed in Table 1. Off-pump patients experienced significantly poorer left ventricular (LV) function (ejection fraction (EF) ≤ 30%) (*p *< 0.05) and significantly higher Euroscores 12.1 ± 1.8 (*p *≤ 0.05). Respiratory problems included chronic obstructive pulmonary disease (COPD) that required active treatment at the time of surgery.

**Table 1:** Preoperative data of patients

Characteristics of the 86 patients studied	Overall population (n = *86*)	Off-pump (n = *40*)	On-pump (n = *46*)	p
Mean age at operation (years)	61.5 ± 8.9	63 ± 12	60 ± 7	0.82
Female sex (%)	10.46	12.50	8.69	0.73
Smoker (%)	27.90	35.00	21.73	0.23
Diabetes (%)	23.25	27.50	19.56	0.38
Hypertension (%)	40.69	50.00	32.60	0.10
Chronic obstructive pulmonary disease (%)	6.97	12.50	2.10	0.06
Mean Left Ventricle				
Ejection Fraction (±SD)	34.2 ± 9.1	28.6 ± 5.8	40.5 ± 7.9	0.032
Mean Euroscore (±SD)	9.7 ± 3.1	12.1 ± 1.8	7.26 ± 1.8	0.022

### Operative characteristics

The operative characteristics of patients are presented in Table 2. There was no significant difference in the number of grafts between the off-pump and on-pump patients (2.03 ± 0.7 vs.1.99 ± 0.6 grafts per patient respectively, *p *= 0.15). Nor were there any significant differences between on-and off pump patients with regard to duration surgery (105 ± 22 vs. 80 ± 25, respectively, *p *= 0.26) or aortic cross-clamp time (21.5 ± 7.6 vs. 20.6 ± 7.5 min, respectively, p = 0.861).

**Table 2:** Intraoperative and postoperative variables.

Characteristics of the 86 patients studied (mean)	Overall population (n = *86*)	Off-pump (n = *40*)	On-pump (n = *46*)	p
Distal anastomosis time (min)	22.75 ± 5.8	20.6 ± 7.5	21.5 ± 7.6	0.861
Duration of surgery (min)	92.50 ± 25	80 ± 25	105 ± 22	0.26
Duration of anesthesia (min)	118.5 ± 28.7	105 ± 19	132 ± 34	0.29
Operating room time (min)	134.5 ± 22.2	124 ± 15	145 ± 26	0.33
Number of grafts/patients	1.99 ± 0.6	1.93 ± 0.7	2.03 ± 0.7	0.15
Extubation time (min)	19.5 ± 10.25	15 ± 9	24 ± 11	0.33
Length of stay in ICU (h)	19 ± 5.2	18 ± 4	20 ± 7	0.69
Length of stay in hospital(days)	7.5 ± 1.5	8 ± 1	7 ± 2	0.48

### Postoperative morbidity

Overall ten (11.62%) overall patients developed peri-operative atrial fibrillation, with no significant difference between the 2 groups. We could not show any significant difference between the off-pump and on-pump patients. Three (7.5%) off-pump patients developed low cardiac output syndrome (LCOS) in the postoperative period compared to 1 (2.1%) on-pump patients (*p *= 0.24). There was no statistically significant difference between the patients with regard to other complications (Table 3).

**Table 3:** Complications after coronary artery bypass grafting after 30 days

Complications of the 86 patients studied	Overall population(%) (n = *86*)	Off-pump(%) (n = *40*)	On-pump(%) (n = *46*)	p
Atrial fibrillation	11.6	17.5	6.5	0.11
LCOS	4.6	7.5	2.1	0.24
Bleeding	3.4	2.5	4.3	0.64
Re-operaton	2.3	2.5	2.1	0.92
Re-ıntubation	3.4	2.5	4.3	0.64
Renal complications	5.8	7.5	4.3	0.53
Pulmonary complications	4.6	5	4.3	0.89
IABP	4.6	7.5	2.1	2.1
30-day mortality	3.4	7.5	0	0.06

LCOS: Low cardiac output syndrome

IABP: Intraaortic balloon pump

As noted in Table 2 the intensive care unit (ICU) stay for off-pump patients was 18 ± 4 h while for on-pump patients it was 20 ± 7 h (*p *= 0.69). The hospital stay was 8 ± 1 days for the off-pump patients and 7 ± 2 days for on-pump patients (*p *= 0.48).

### Postoperative mortality

We defined postoperative mortality as death within the 30 days following the operation (Table 3). There were three (7.5%) deaths in the off-pump patients compared to no death in the on-pump patients (*p *= 0.06) within 30 days postoperatively. The three of off-pump deaths included two due to cardiac causes, one due to multi-organ failure (MOF).

## Discussion

Based on our findings in this retrospective comparative study, use of the off-pump technique for myocardial revascularization in extreme preoperative high risk (Euroscore ≥ 10, EF < 30%) patients reduces the incidence of perioperative morbidity and mortality, ICU stay and other complications when compared to on-pump patients.

European and US institutional data demonstrate that patients undergoing CABG are progressively older and have a worse cardiac status and a higher incidence of systemic co-morbidities. It seems highly likely that this trend will increase and that high-risk patients will represent a greater proportion of patients treated by cardiac surgeons [16-21].

The initial application of the off-pump technique in the early nineties was mainly directed to highly selected and relatively low-risk surgical patients [22]. Since then there has been a growing body of evidence suggesting many potential advantages of the off-pump technique over the conventional CPB in different groups of high-risk patients [23,24].

In this setting the standard surgical strategy is often inappropriate and carries substantial operative risks. To date, however, to date few reports have focused on the results of off-pump versus on-pump conventional surgery in high-risk patients. In patients with acute or chronically ischemic myocardium and poorly functioning left ventricles, off-pump and on-pump surgical revascularization have been shown to improve survival, improve functional status or control ischemic symptoms, and diminish the prevalence of sudden cardiac deaths caused by arrhythmias [23-25]. Moreover, methodological issues and the heterogeneity of reported results have precluded any definitive conclusion on the possibility that off-pump surgery can reduce the operative risk of complex CABG patients [23-25].

Our study is a non-randomized comparative retrospective study of patients who underwent first-time isolated coronary bypass surgery on- or off-pump in our center. Preoperative variables in the overall patients showed little variation between the on-pump and off-pump patients except for EF (*p *= 0.032) and for Euroscore (*p *= 0.022) which were significantly lower in off-pump patients. The similar number of anastomoses performed in the on-pump and off-pump patients. New onset of atrial fibrillation was reduced in the on-pump patients in our series but not significantly (*p *= 0.11). There were no significant differences in the incidence of perioperative LCOS, renal complications, pulmonary complications and intraaortic ballon pump (IABP) using the off-pump vs. the on-pump technique. Similar fidings were also noted for intubation time, intensive care unit stay and hospital stay.

Data from the Euroscore project indicate that patients in the highest risk groups who undergo conventional surgery can have hospital mortality as high as 11.2% [8]. The present series describes our experience in the treatment of high-risk CABG patient and compares patients assigned to on-pump vs. off-pump revascularization. Overall mortality (3 of 86 patients, 3.4%) was one of the lowest reported in patients of this type. In contrast to other publications, the hospital mortality in our series was not significantly different between off-pump and on-pump patients. We agree with other authors that the improved results may be attributed to advances in myocardial protection, surgical technique, and perioperative care.

In conclusion, our data suggest that the adoption of off-pump CABG does not confer significant clinical advantages in all high-risk patients. This review supports the off-pump coronary revascularization, which may represent an alternative approach for treating patients with Euroscore ≥ 10 who have left ventricular function ≤ 30%.
